# The Past, Present, and Future of Dyslipidemia: A Narrative Review

**DOI:** 10.7759/cureus.106258

**Published:** 2026-04-01

**Authors:** Prabhash C Manoria

**Affiliations:** 1 Cardiology, Manoria Heart and Critical Care Hospital, Bhopal, IND

**Keywords:** atherogenesis, cacs, cardiovascular disease, dyslipidemia, lipoprotein

## Abstract

Cardiovascular disease (CVD) remains the leading global cause of death, with dyslipidemia as its most modifiable driver of atherosclerotic CVD. The burden is especially severe in India, where early-onset disease and a distinct lipid profile, marked by elevated triglycerides, low high-density lipoprotein cholesterol (HDL-C), and small dense low-density lipoprotein (sdLDL) particles, contribute to increased lifetime risk.

The pathophysiology of atherosclerosis has evolved from the cholesterol-centric model to the response-to-retention hypothesis, highlighting the role of apolipoprotein B (ApoB)-containing lipoproteins in initiating vascular inflammation and plaque formation. Advances in lipid science have revealed complex interactions between lipid metabolism, endothelial dysfunction, and immune activation.

The Lipid Association of India 2023 guidelines recommend lifetime risk-based assessment and early intervention using coronary artery calcium scoring (CACS), carotid and femoral plaque imaging, and ankle-brachial index (ABI) to refine risk stratification.

Therapeutic options have expanded beyond statins to include ezetimibe, PCSK9 inhibitors, bempedoic acid, and RNA-based agents, offering deeper and safer lipid reduction. Emerging therapies targeting ANGPTL3, liver X receptor (LXR) β, and PPAR-β/δ pathways promise further innovation in lipid modulation.

These developments mark a shift toward precision lipidology, integrating genomics, imaging, and long-acting therapeutics to enable early, aggressive, and individualized prevention. As India faces a rising tide of cardiovascular events in younger populations, the future of lipid management lies in personalized, multimodal strategies that address both traditional and residual risk, aiming for sustained cardiovascular protection and improved public health outcomes.

## Introduction and background

Cardiovascular disease (CVD) remains the foremost cause of global morbidity and mortality, responsible for nearly one-third of all deaths. Among the metabolic determinants of this epidemic, dyslipidemia stands as the most pivotal and modifiable risk factor, driving the progression of atherosclerotic cardiovascular disease (ASCVD). Despite significant advances in preventive cardiology, the worldwide burden of CVD continues to escalate. Between 2025 and 2050, global CVD prevalence is projected to nearly double, from 598 million to 1.14 billion cases, with cardiovascular mortality rising from 20.5 million to 35.6 million deaths, representing a 73.4% (2.9% year-on-year) increase, primarily due to population ageing and persistent metabolic risk exposure [1]. India contributes substantially to this crisis, with an age-adjusted CVD death rate of 349 per 100,000 in men and 265 per 100,000 in women, exceeding the global average. Indians experience cardiovascular events almost a decade earlier than Western populations, and coronary artery disease prevalence has risen from 1%-2% in the 1960s to 10%-12% in urban and 4%-6% in rural regions. The incidence of these conditions is rising alongside diabetes (10%-12%), hypertension (30%), dyslipidemia (25%-30%), sedentary lifestyle (41%), and obesity (20%-25%) [2]. Indians face a particularly high burden of ASCVD risk due to the interplay of obesity, diabetes, and chronic kidney disease (CKD), often at lower body weights than Western populations. While obesity prevalence is lower in Indians (≈21%) than in Americans (≈41%), Indians develop diabetes (7.7%) and CKD (13.24%) at younger ages, amplifying ASCVD risk [3,4].

Dyslipidemia, the biochemical hallmark of ASCVD, actively drives atherogenesis through endothelial dysfunction, oxidative modification of low-density lipoprotein (LDL), foam-cell formation, and plaque instability [5]. South Asians commonly display an atherogenic dyslipidemic phenotype, which includes high triglycerides, low high-density lipoprotein cholesterol (HDL-C), and small dense LDL (sdLDL), contributing to early-onset ASCVD and residual risk despite statin therapy. Beyond conventional lipid indices, emerging markers such as non-HDL-C, apolipoprotein B (ApoB), and the atherogenic index of plasma offer superior risk prediction, particularly in high-risk and dyslipidemic populations [5].

Whereas our previous work primarily focused on evaluating LDL-C-lowering therapies and their efficacy in high-risk populations [6], the present review expands the scope to integrate pathophysiology and emerging precision lipid-lowering strategies. This review revisits the evolution of lipid research, delineates current global and Indian trends, and emphasizes recent advances in lipid-lowering strategies, incorporating recommendations from the Lipid Association of India (LAI) 2023 guidelines to guide the future of precision lipidology in ASCVD prevention.

## Review

Materials and methods

This manuscript is a narrative review summarizing current evidence on dyslipidemia, ASCVD, and evolving lipid-lowering strategies, with relevance to the Indian population. A comprehensive literature search was conducted using electronic databases, including PubMed and Google Scholar, for studies published up to 2025. Keywords used in various combinations included “dyslipidemia,” “atherosclerotic cardiovascular disease,” “lipid metabolism,” “ApoB lipoproteins,” “precision lipidology,” “India,” and “lipid-lowering therapy.” Relevant peer-reviewed articles, including clinical trials, observational studies, meta-analyses, and guideline documents, were reviewed. Particular emphasis was placed on major international guidelines and the LAI 2023 recommendations, as well as on studies highlighting the unique lipid phenotype and cardiovascular risk profile of South Asian populations. Additional relevant articles were identified through manual screening of the reference lists of selected publications. Studies were selected based on their relevance to ASCVD risk assessment, lipid biology, and established or emerging lipid-lowering therapies. The findings were synthesized qualitatively to provide a comprehensive overview of current evidence, with a focus on risk stratification, advances in lipid science, and evolving therapeutic strategies relevant to clinical practice.

Historical perspective

The lipid theory of atherogenesis, proposed more than a century ago, remains the cornerstone of atherosclerosis prevention and treatment, having evolved from the concept of lipids as passive substrates to their recognition as complex immune-metabolic regulators and pivotal diagnostic and therapeutic targets [7]. Over the decades, this foundational theory has expanded through discoveries linking lipid metabolism to vascular inflammation, endothelial dysfunction, and immune activation, transforming the understanding of atherosclerosis from a simple degenerative process to a chronic inflammatory and metabolic disorder.

A major conceptual advance came with the formulation of the response-to-retention hypothesis, which superseded the earlier response-to-injury model. This paradigm identified the subendothelial retention of ApoB-containing lipoproteins, including LDL, lipoprotein(a) (Lp(a)), and triglyceride-rich remnants, as the initiating step in atherogenesis. These lipoproteins traverse the endothelial barrier primarily via receptor-mediated transcytosis and become trapped by proteoglycans within the arterial intima through electrostatic binding to ApoB’s basic amino acid residues. Once retained, they undergo oxidation and aggregation, releasing pro-inflammatory mediators that trigger endothelial activation, monocyte recruitment, and macrophage transformation into foam cells [8]. This mechanistic framework established the cholesterol hypothesis, which recognized hypercholesterolemia as a central and modifiable cause of atherosclerosis. The identification of both ApoB-100 and ApoB-48 isoforms as equally atherogenic reinforced the concept that arterial lipid retention - not endothelial injury alone - drives plaque formation, laying the groundwork for lipid-lowering therapy as the cornerstone of ASCVD prevention [8].

Parallel to these mechanistic insights, the therapeutic approach to atherosclerosis underwent a transformative evolution. Early management centered on risk factor modification, such as dietary changes, smoking cessation, and exercise, complemented by pharmacologic interventions including aspirin, statins, β-blockers, and angiotensin-converting enzyme (ACE) inhibitors, which together reduced cardiovascular morbidity and mortality by nearly 50% [9]. Subsequently, the pursuit of non-invasive therapies led to innovative modalities such as enhanced external counterpulsation (EECP), shown in the MUST-EECP trial to improve myocardial perfusion and angina through diastolic flow augmentation; extracorporeal cardiac shock wave therapy (ECST), promoting angiogenesis; and low-energy laser irradiation (LELI), which demonstrated cardioprotective and mitochondrial benefits. Experimental advances in ultrasound-enhanced thrombolysis further broadened non-invasive management options for ischemic heart disease and stroke [9].

Pathophysiology

Overview of Lipoprotein Pathways

Lipid transport in the human body occurs through three interconnected systems: exogenous, endogenous, and reverse cholesterol transport pathways, which collectively maintain lipid homeostasis and influence atherogenesis [10]. In the exogenous pathway, dietary triglycerides, cholesterol, and phospholipids absorbed in the intestine are packaged into chylomicrons, which deliver triglycerides to peripheral tissues. The resulting chylomicron remnants are subsequently cleared by hepatic receptors. The endogenous pathway begins in the liver, where newly synthesized triglycerides and cholesterol are secreted as very-low-density lipoproteins (VLDLs). Progressive hydrolysis by lipoprotein lipase (LPL) and hepatic lipase transforms VLDL into LDL, the principal cholesterol carrier, delivering cholesterol to peripheral tissues via the LDL receptor. In contrast, HDL mediates reverse cholesterol transport, a critical anti-atherogenic process. Apolipoprotein A1 (ApoA-1) promotes cholesterol efflux from macrophages through ABC-A1 and ABC-G1 transporters, while lecithin-cholesterol acyltransferase (LCAT) esterifies free cholesterol to form mature HDL. Cholesteryl esters are then delivered back to the liver directly via SR-BI receptors or indirectly through cholesteryl ester transfer protein (CETP) exchange with ApoB-containing lipoproteins [10]. Disruption of any of these pathways, especially excess LDL or impaired HDL function, predisposes to atherogenesis.

Lipid Metabolism and Atherogenesis

Lipid metabolism orchestrates a complex interplay of biochemical and biomechanical processes that initiate and accelerate atherogenesis. The endothelium, once considered a passive barrier, is now recognized as a dynamic organ that senses mechanical forces and regulates lipid flux, oxidative stress, and inflammation. Within endothelial membranes, cholesterol- and sphingolipid-enriched microdomains, known as lipid rafts and caveolae, govern nitric oxide (NO) signaling and mechano-transduction. The structural protein caveolin-1 modulates endothelial nitric oxide synthase (eNOS) activity, while its dysregulation leads to oxidative stress and LDL transcytosis, fostering plaque formation. Excessive cholesterol accumulation stiffens membranes, whereas oxidation of LDL disrupts caveolae, decreases NO production, and triggers endothelial activation. Concurrently, ceramide generation via sphingomyelinase increases endothelial permeability and apoptosis, amplifying lipid retention and vascular inflammation [11].

Beyond cholesterol, triglyceride-rich lipoproteins (TRLs) play a direct atherogenic role. Derived from chylomicrons and VLDL, these particles undergo partial hydrolysis by LPL to form cholesterol-enriched remnants that readily infiltrate the arterial wall. Once retained, TRL remnants induce endothelial activation, upregulate vascular cell adhesion molecule-1 (VCAM-1) and intercellular adhesion molecule-1 (ICAM-1), and promote foam-cell formation via macrophage uptake. Surface apolipoproteins ApoC-III and ApoB-100 enhance oxidative stress, inflammation, and thrombosis. Moreover, triglyceride enrichment of LDL through CETP-mediated lipid exchange generates sdLDL, which is more atherogenic due to increased oxidation susceptibility and prolonged circulation. Elevated triglycerides also stimulate smooth muscle cell proliferation through platelet-derived growth factor (PDGF) signaling, further contributing to plaque growth and instability [12].

Therefore, cholesterol-driven endothelial dysfunction and triglyceride-mediated lipoprotein remodeling act together to promote lipid deposition, inflammation, and vascular remodeling - the fundamental triad of atherogenesis.

LAI 2023 guideline recommendations

The LAI 2023 Consensus Statement provides the most comprehensive, India-specific recommendations for cardiovascular risk assessment and lipid management, incorporating new global evidence, local epidemiology, and subclinical atherosclerosis data. India faces an unprecedented cardiovascular epidemic: over 50% of coronary artery disease deaths occur before age 50, and 25% of myocardial infarctions (MIs) occur before 40 years, prompting the LAI to prioritize lifetime ASCVD risk rather than traditional 10-year models [13].

Updated Risk Stratification and Framework

The LAI 2023 guidelines present an updated cardiovascular risk assessment algorithm, along with corresponding recommendations for LDL-C lowering. In the Indian population, the emphasis is placed on lifetime risk reduction, underscoring the importance of initiating preventive interventions at an early stage. ASCVD risk is stratified into major risk factors, high-risk features, and risk modifiers. Major risk factors include advancing age (≥45 years in men, ≥55 years in women), tobacco use, hypertension, and low HDL-C. High-risk features encompass family history of premature ASCVD, CKD Stage 3B-4, elevated ApoB, markedly raised single risk factors, Lp(a) ≥50 mg/dL, metabolic syndrome, significant non-alcoholic fatty liver disease (NAFLD) fibrosis, and subclinical atherosclerosis (coronary artery calcium score (CACS) 1-99, <75th percentile). Risk modifiers include intermediate elevations of Lp(a), impaired fasting glucose, central obesity, elevated high-sensitivity C-reactive protein (hsCRP), hypertriglyceridemia, chronic inflammatory diseases, adverse pregnancy-related conditions, high polygenic risk score, air pollution exposure, and HIV infection. Recognizing that pooled cohort equations and Western calculators underestimate risk in Indians, the LAI now advocates for Indian-specific lifetime ASCVD risk assessment using the QRISK3-lifetime ASCVD risk calculator. The statement emphasizes early identification of “at-risk” individuals, particularly those with a family history of premature ASCVD, diabetes, metabolic syndrome, or elevated triglycerides (≥150 mg/dL). These updates ensure earlier therapeutic intervention, even in younger adults, to mitigate lifelong exposure to atherogenic lipoproteins. These guidelines bridge the gap between conventional risk algorithms and the distinct cardiometabolic profile of South Asians, promoting precision-based cardiovascular prevention [13].

According to the LAI 2023 guidelines [13], ASCVD risk is stratified into six categories, as follows: (a) Low risk requires all of the following: 0-1 major ASCVD risk factor, LDL-C 100-129 mg/dL, non-HDL-C 130-159 mg/dL, and a lifetime ASCVD risk <30% (assessed using the QRISK lifetime calculator); (b) Moderate risk is defined by any one of: 2 major risk factors; LDL-C 130-159 mg/dL; non-HDL-C 160-189 mg/dL; low-risk status plus ≥1 risk modifier; or lifetime risk >30%; (c) High risk includes ≥3 major risk factors; LDL-C 160-189 mg/dL; non-HDL-C 190-219 mg/dL; diabetes with 0-1 major risk factor; 2 major risk factors plus ≥1 modifier; or the presence of any high-risk feature; (d) Very high risk comprises diabetes with target organ damage, diabetes plus ≥2 major risk factors, CACS 100-299 or >75th percentile (if CACS 1-99), ≥2 high-risk features, established ASCVD (including carotid, femoral, or coronary plaque, or ankle-brachial index (ABI) <0.9), or heterozygous familial hypercholesterolemia (FH)/LDL-C ≥190 mg/dL; and (e) Extreme risk is subdivided into: Category A (ASCVD plus ≥1 high-risk feature, CACS ≥300, or homozygous FH); Category B (ASCVD with ≥1 very high-risk feature, recurrent acute coronary syndrome, polyvascular disease, or homozygous FH); and Category C (recurrent ASCVD events despite LDL-C around 30 mg/dL).

Family History as a High-Risk Feature

A family history of premature ASCVD, defined as an event in male first-degree relatives <55 years and female first-degree relatives <65 years, or before menopause, is now recognized as an independent high-risk feature. It reflects both genetic and environmental susceptibility and warrants early screening and aggressive risk factor modification. Clinicians are encouraged to document ASCVD events and their age of onset in all first-degree relatives and, when possible, in grandparents, aunts, and uncles. The number of affected relatives directly correlates with an individual’s lifetime ASCVD risk. This update aligns with the European Society of Cardiology (ESC) and European Atherosclerosis Society (EAS) recommendations and underscores the importance of detailed familial risk assessment in Indian clinical practice [13].

Subclinical Atherosclerosis as a Risk Modifier

Traditional risk scores provide population-based probabilities and may overlook individuals with early or silent atherosclerosis. The LAI 2023 guidelines emphasize the use of subclinical atherosclerosis detection as a superior method for refining risk stratification. Tools such as CACS and carotid or femoral plaque imaging using computed tomography (CT) or ultrasound offer direct visualization of atherosclerotic burden and provide stronger predictive value than conventional clinical markers. The ABI also remains a useful, low-cost adjunct for identifying peripheral arterial disease (PAD) and enhancing overall ASCVD risk estimation [13].

Coronary Artery Calcium Score (CACS)

The CACS, quantified by the Agatston method, serves as a robust and accessible marker of total coronary plaque burden. A CACS ≥100 indicates high to very high risk, warranting initiation or intensification of statin therapy and consideration of low-dose aspirin. A CACS = 0 conveys a low short-term event risk (“power of zero”) but should not override clinical judgment in patients with multiple risk factors or comorbidities. A CACS of 1-99, especially values above the 75th percentile for age, sex, and ethnicity, or in individuals aged <45 years, indicates high lifetime ASCVD risk and justifies preventive therapy.

Evidence from major studies, such as the Multi-Ethnic Study of Atherosclerosis (MESA), Coronary Artery Risk Development in Young Adults (CARDIA), and pooled Indian cohorts, confirms a dose-response relationship between CACS and ASCVD events. Individuals with CACS ≥100 have 10%-15% 10-year event rates, exceeding treatment thresholds for pharmacologic intervention [13].

Carotid and Femoral Artery Imaging

Multiple studies affirm that non-obstructive carotid and femoral plaques independently predict ASCVD events. In the CAFES-CAVE study (n = 14,300; 10-year follow-up), participants with plaques ≥1 mm and irregular echogenicity experienced a 39.1% event rate. Similarly, another cohort showed event rates rising with the number of arteries involved: 5.7% events (no plaque), 12%-13% events (one to two arteries), and 22%-23% events (all three arteries). The Aragon Workers’ Health Study found that femoral plaques (prevalence 54%) predicted risk better than carotid plaques (34%) or coronary calcification (38%). The ARIC study further demonstrated that including plaque data with CACS reclassified 23% of individuals into higher-risk categories. Although carotid/femoral ultrasound is inexpensive and radiation-free, its accuracy depends on operator expertise and standardized protocols. Quantitative parameters, such as plaque area, height, and volume, enhance precision, while MRI-based plaque characterization remains limited by cost and availability [13].

Ankle-Brachial Index (ABI)

A low ABI (<0.9) signifies PAD and predicts nearly two-fold higher mortality. The ABI Collaboration study (45,000 participants) demonstrated that including ABI data showed risk reclassification in 36% of women and 19% of men when ABI data was added to the Framingham risk score. The LAI 2023 considers an ABI <0.9 as established ASCVD, recommending LDL-C targets <50 mg/dL, equivalent to secondary prevention thresholds [13].

Implications for the Indian Population

The LAI 2023 identifies several imaging and clinical features that warrant intensive LDL-C lowering (LDL-C <50 mg/dL), such as CACS ≥100; CACS 1-99 and above the 75th percentile for age, sex, and race; non-stenotic coronary, carotid, or femoral plaques (LAI 2023); stenotic (>50%) coronary, carotid, or femoral plaques; or ABI <0.9 (as per LAI 2016).

Patients with CACS ≥300 fall into the extreme-risk category, with an LDL-C goal <50 mg/dL (optional LDL-C goal ≤30 mg/dL), while those with CACS 1-99 (<75th percentile) are classified as high risk (goal: LDL-C <70 mg/dL). Recognizing that Indians develop ASCVD a decade earlier than Western populations, these thresholds are intentionally more aggressive. Even minimal calcification or early plaque in younger adults signals high lifetime ASCVD risk, supporting early statin initiation and comprehensive risk factor management. CACS testing is simple, non-invasive, radiation-free, and affordable in India [13].

Recommendation for Subclinical Atherosclerosis Assessment

The LAI 2023 Consensus recommends assessment for subclinical atherosclerosis in individuals aged ≥30 years when treatment decisions remain uncertain after conventional risk scoring and clinical evaluation. This is particularly advised for: (a) those classified as moderate- or high-risk by the LAI algorithm; (b) individuals with a family history or uncertain history of premature ASCVD; (c) suspected or confirmed FH; (d) persons with multiple ASCVD risk factors; (e) statin-intolerant or treatment-reluctant patients.

These recommendations are intentionally more aggressive than Western guidelines, reflecting the high baseline risk and earlier onset of ASCVD among Indians, thereby emphasizing the need for early detection and preventive intervention [13].

The ASCVD Risk Estimation Calculator

The LAI 2023 had recommended the use of the QRISK3-lifetime cardiovascular risk calculator (https://qrisk.org/lifetime/) for estimating long-term cardiovascular risk in Indian adults [13]. This tool integrates multiple clinical parameters, including blood pressure, smoking status, lipid profile, and comorbid conditions such as diabetes, CKD, angina, rheumatoid arthritis, migraine, and hypercholesterolemia, to generate a personalized risk score [14]. The calculated risk aids clinicians in tailoring preventive strategies, including lifestyle modifications and pharmacotherapy, especially for individuals at borderline or intermediate risk. Currently, the LAI has designed an ASCVD risk calculator to predict the occurrence of a first acute coronary syndrome event, and the PRECAD study is ongoing.

Therapeutic management

Lipid Targets and Treatment Hierarchy

The LAI 2023 recommends a comprehensive, target-based approach to lipid management. The recommendations for lipid targets are based on ASCVD risk. For low-risk individuals, LDL-C <100 mg/dL, non-HDL-C <130 mg/dL, and ApoB <90 mg/dL are advised. Moderate-risk targets are similar, with optional intensification to LDL-C <70 mg/dL and non-HDL-C <100 mg/dL. High-risk patients should aim for LDL-C <70 mg/dL, non-HDL-C <100 mg/dL, and ApoB <80 mg/dL, while very high-risk patients require LDL-C <50 mg/dL, non-HDL-C <80 mg/dL, and ApoB <65 mg/dL. In extreme-risk categories, LDL-C and non-HDL-C targets are more aggressive, ranging from <50 to 10-15 mg/dL for LDL-C, depending on subcategory, with corresponding reductions in non-HDL-C and ApoB. LDL-C remains the primary therapeutic target, while non-HDL-C serves as a co-primary target, and ApoB as a secondary target. Non-HDL-C, measurable in the non-fasting state, should be interpreted alongside LDL-C to guide therapy. LDL-C represents the cholesterol content of LDL particles, whereas non-HDL-C reflects the cholesterol in all atherogenic lipoproteins. Since each atherogenic particle, including LDL, IDL, VLDL remnants, and Lp(a), contains one molecule of ApoB, its concentration accurately indicates the total number of atherogenic particles. Multiple studies have shown ApoB to be a stronger predictor of ASCVD risk than LDL-C or non-HDL-C, though these measures often concur in individuals with normal triglyceride levels. Accordingly, the LAI endorses ApoB as a secondary therapeutic target [13].

Global Perspectives on ASCVD Management

Contemporary global guidelines converge on the principle that lower LDL-C is better and earlier is optimal, yet differ in their approach to risk stratification and therapeutic intensification. The ESC/EAS 2025 focused update on dyslipidemia management guidelines reinforces a risk-based strategy using SCORE2/SCORE2-OP (Systematic Coronary Risk Evaluation 2/Systematic Coronary Risk Evaluation 2-Older Persons) for 10-year ASCVD risk estimation, advocating LDL-C targets of <55 mg/dL for very high-risk and <70 mg/dL for high-risk individuals, with combination therapy (statin + ezetimibe ± PCSK9 inhibitor) recommended during index hospitalization for acute coronary syndromes to achieve rapid goal attainment. Non-statin agents, such as bempedoic acid, inclisiran, and ANGPTL3 inhibitors, are incorporated for patients with statin intolerance or persistent elevation despite maximal therapy [15]. The ACC/AHA 2025 ACS (Acute Coronary Syndrome) guideline emphasizes early, aggressive lipid lowering, lowering the intensification threshold to LDL-C 55-69 mg/dL, and endorses simultaneous initiation of high-intensity statin plus ezetimibe at discharge, with PCSK9 inhibitors or bempedoic acid considered for high-risk subgroups. Importantly, very low LDL-C levels are deemed safe, supporting the “strike early and strong” paradigm [16]. The AACE 2025 guideline adopts a pragmatic stance, recommending an LDL-C goal of <70 mg/dL for adults with ASCVD or those at high risk. It conditionally supports the use of PCSK9 inhibitors and bempedoic acid for statin-intolerant patients, or for individuals on a maximal statin dose who have not achieved target LDL-C levels (<70 mg/dL), while cautioning against the routine use of niacin and mixed omega-3 formulations [17]. Across all guidelines, ApoB and non-HDL-C are recognized as secondary targets, and Lp(a) measurement is increasingly advised as a risk enhancer. Collectively, these updates underscore a global shift toward personalized, combination-based therapy, leveraging novel agents to achieve deeper and sustained lipid lowering for optimal cardiovascular protection.

Current Therapies for Dyslipidemia

The management of dyslipidemia has evolved remarkably over the past three decades, anchored initially by statin therapy and later expanded through multiple non-statin lipid-lowering agents. Statins, competitive inhibitors of HMG-CoA reductase, remain the cornerstone of therapy by reducing hepatic cholesterol synthesis and upregulating hepatic LDL receptors, resulting in substantial LDL-C reduction and proven reductions in major adverse cardiovascular events (MACE). In primary prevention, the WOSCOPS trial (pravastatin) demonstrated a 31% reduction in non-fatal MI and CHD deaths, while AFCAPS/TexCAPS (lovastatin) showed a 37% reduction in first major coronary events. The ASCOT-LLA study (atorvastatin) demonstrated a 36% reduction in non-fatal MI and CHD deaths, and JUPITER (rosuvastatin) reported 50% LDL-C and 37% hs-CRP reductions, translating into a 44% lower composite risk of MI, stroke, and cardiovascular death. HOPE-3 (rosuvastatin) showed a 24% reduction in composite cardiovascular outcomes across multiple ethnicities. In secondary prevention, high-intensity statins (PROVE-IT TIMI 22, TNT) achieved 16%-22% further event reductions, while the 4S, CARE, and LIPID trials demonstrated mortality benefits with simvastatin and pravastatin post-MI. Indian data from IRIS and CURE-ACS confirmed up to 50% LDL-C reduction with rosuvastatin and atorvastatin. The CTT meta-analysis established that every 1 mmol/L LDL-C reduction lowers major vascular events by 22%, and pharmacokinetic data showed that Asian Indians achieve higher statin plasma levels, warranting lower rosuvastatin starting doses (5 mg) [18,19].

Non-statin therapies now complement or substitute statins in specific contexts. Ezetimibe, an NPC1L1 inhibitor, produced an additional 18% LDL-C reduction and 2% absolute event reduction in IMPROVE-IT (n = 18,144) when added to simvastatin post-ACS. PCSK9 inhibitors, evolocumab and alirocumab, showed significant outcome benefits: FOURIER (n = 27,564; 13.9% Asia-Pacific) achieved a 59% LDL-C reduction and 15% MACE decline, while ODYSSEY OUTCOMES (n = 18,924; 521 Indians) demonstrated a 15% event reduction post-ACS. Bempedoic acid, an ATP-citrate lyase inhibitor, provided 15%-28% LDL-C reductions in the CLEAR trials and a 13% MACE reduction in CLEAR Outcomes (HR 0.87; 95% CI 0.79-0.96; p = 0.004). Icosapent ethyl (EPA), studied in REDUCE-IT (n = 8,179; 262 Indians), achieved an 18% triglyceride and 25% MACE reduction. Fibrates, acting via PPAR-α activation, lower triglycerides and modestly raise HDL-C but failed to reduce mortality in FIELD and ACCORD, except in severe hypertriglyceridemia. Niacin increased HDL-C but showed no cardiovascular benefit and more adverse events in HPS2-THRIVE. Novel agents, such as inclisiran, mipomersen, volanesorsen, lomitapide, and evinacumab, demonstrate 30%-50% LDL-C reductions in early-phase trials, though Indian cohort data remain limited [19].

Recent Advances in Lipid Management

Therapeutic innovation in dyslipidemia has rapidly progressed beyond statins, introducing agents that target novel molecular pathways to optimize lipid lowering, improve adherence, and address residual cardiovascular risk. The PCSK9 pathway has emerged as a major focus, with monoclonal antibodies, such as evolocumab and alirocumab, demonstrating potent LDL-C reductions. In the FOURIER trial, evolocumab reduced LDL-C by 59%, resulting in a 15% decline in the composite primary endpoint and a 20% reduction in key secondary endpoints over 2.2 years, with continued event reduction even at extremely low LDL-C levels (<0.2 mmol/L or 7.7 mg/dL), without safety concerns, as confirmed by the EBBINGHAUS sub-study. Alirocumab similarly reduced composite cardiovascular outcomes by 15% over 2.8 years in post-ACS patients in the ODYSSEY OUTCOMES trial.

Inclisiran, a small-interfering RNA (siRNA) that inhibits hepatic PCSK9 synthesis, offers durable LDL-C lowering with infrequent dosing - initially, at three months, and then every six months. In ORION-10 and ORION-11, inclisiran reduced LDL-C by approximately 50%-52%, with a favorable safety profile limited to mild injection-site reactions.

Lerodalcibep, a third-generation PCSK9 inhibitor and recombinant fusion protein, achieved a 58.6% LDL-C reduction at 24 weeks in the LIBerate-HeFH trial, along with reductions in ApoB and Lp(a), and a 62% LDL-C reduction at 52 weeks in the LIBerate-CVD trial, with over 90% of participants reaching LDL-C <55 mg/dL. Although its efficacy is modest in homozygous FH due to absent LDL receptor function, overall tolerability remains favorable.

Additionally, bempedoic acid, a liver-specific ATP-citrate lyase inhibitor acting upstream of HMG-CoA reductase, lowered LDL-C by 15%-28% across the CLEAR clinical program, and also reduced non-HDL-C, ApoB, total cholesterol, and hs-CRP. In CLEAR Outcomes, it achieved a 13% reduction in major cardiovascular events over 3.4 years in statin-intolerant patients, with mild hyperuricemia and gout as the main reported adverse effects, but an overall favorable safety profile [20,21].

CETP inhibitor trials have failed to improve outcomes by raising HDL-C, but the new molecule, Obicetrapid, has shown a 41.5% decrease in LDL-C and a 54% decrease in Lp(a) [22]. The PREVAIL cardiovascular outcome trial is ongoing [23]. Pelacarsen, an antisense oligonucleotide (ASO) targeting Apo(a), is being evaluated in the HORIZON trial [24]. Three small interfering RNAs - Olpasiran, Lepodisiran, and Zerlasiran - are undergoing Phase III trials and are expected to reduce Lp(a) by 90%-95% [25]. Muvalaplin, an oral molecule, has also shown a decrease in Lp(a) by 60%-65% in the Phase II KRAKEN trial and is currently undergoing evaluation in a Phase III trial [26].

Emerging Therapies and Future Vision

The ANGPTL3 inhibition represents a novel strategy in lipid modulation by targeting hepatic angiopoietin-like protein 3, a key regulator of lipoprotein and endothelial lipase activity. Pharmacologic blockade of ANGPTL3 has shown reductions in triglycerides, LDL-C, and ApoB, offering dual lipid-lowering and anti-atherogenic benefits. In parallel, nuclear receptor-based therapies are advancing, with selective liver X receptor (LXR) modulators aimed at enhancing reverse cholesterol transport via upregulation of ABCA1 and ABCG1. While full LXR agonism is limited by hepatic lipogenesis and hypertriglyceridemia, newer agents, such as XL-652 and IMB-808, focus on selective LXR-β activation to retain vascular benefits while minimizing metabolic side effects. Additionally, selective PPARβ/δ agonists, like seladelpar (MBX-8025), have demonstrated promising lipid-lowering effects, reducing LDL-C, non-HDL-C, triglycerides, and ApoB, alongside improved insulin sensitivity and anti-inflammatory potential, positioning them as complementary agents to statins or RNA-based therapies [20,21].

Gene editing using CRISPR technology is emerging as a transformative approach. By permanently inactivating PCSK9 in hepatocytes, this strategy aims for a single-dose, lifelong LDL-C reduction, particularly valuable for heterozygous FH. The HEART-1, an ongoing open-label, ascending-dose, phase-1b trial and the first-in-human study of VERVE-101 (an in vivo adenine base-editing therapy), reported encouraging interim results, showing up to 55% LDL-C reduction sustained for six months after one infusion, with marked PCSK9 suppression. Preclinical studies in primates have shown durable LDL-C lowering for over two years without off-target edits [27]. If proven safe and effective, CRISPR-based therapies could redefine lipid management by offering a one-time intervention with sustained LDL-C control, complementing existing pharmacologic options.

The future of lipid management is shifting toward multimodal, precision-based therapy, integrating RNA therapeutics, nuclear receptor modulation, and gene-silencing technologies. In patients unable to achieve LDL-C goals or with statin intolerance, PCSK9 inhibition (mAbs or siRNA) and adenosine triphosphate-citrate lyase inhibitors already provide safe, durable LDL-C reductions with proven or emerging outcome benefits [20,21]. Looking forward, LXR-β-selective agents, PPARβ/δ modulators, and next-generation RNA therapeutics targeting ANGPTL3 or ApoC-III may redefine treatment paradigms, aiming for deeper lipid lowering, inflammation resolution, and plaque regression, with enhanced adherence and long-term cardiovascular protection.

## Conclusions

ASCVD continues to pose a disproportionate burden globally and in India, with dyslipidemia remaining the pivotal, modifiable risk factor. Advances in lipid science have transformed our understanding from a simple cholesterol-centric model to a complex interplay of immune, metabolic, and genetic pathways. The emergence of potent non-statin therapies, including PCSK9 inhibitors, bempedoic acid, ANGPTL3 and ApoC-III inhibitors, and RNA-based therapeutics, has expanded treatment possibilities, enabling deeper and safer lipid lowering. The Lipid Association of India 2023 Guidelines now advocate early, aggressive, and individualized lipid management based on lifetime ASCVD risk, reflecting India’s unique epidemiologic profile.

Looking ahead, precision lipidology, integrating genomics, imaging, and long-acting therapeutics, will redefine cardiovascular prevention. A future where lipid modulation is personalized, sustained, and universally accessible is now within reach, marking a new era in the global fight against ASCVD.

## References

[1] Chong B, Jayabaskaran J, Jauhari SM (2025). Global burden of cardiovascular diseases: projections from 2025 to 2050. Eur J Prev Cardiol.

[2] Loitongbam L, Surin WR (2024). The rising burden of cardiovascular disease and thrombosis in India: an epidemiological review. Cureus.

[3] Kumar A, Shariff M (2019). Atherosclerostic cardiovascular disease risk score: are Indians underestimating the risk of cardiovascular disease?. Indian Heart J.

[4] Talukdar R, Ajayan R, Gupta S (2025). Chronic kidney disease prevalence in India: a systematic review and meta-analysis from community-based representative evidence between 2011 to 2023. Nephrology (Carlton).

[5] Gaggini M, Gorini F, Vassalle C (2022). Lipids in atherosclerosis: pathophysiology and the role of calculated lipid indices in assessing cardiovascular risk in patients with hyperlipidemia. Int J Mol Sci.

[6] Manoria PC, Manoria P, Shrivastava RK, Parashar SK (2020). Dyslipidemia and atherosclerotic cardiovascular disease: flash back and vision ahead. Am J Intern Med.

[7] Kotlyarov SN (2024). Place of lipid theory in history of study of atherosclerosis. Russian Medical Biological Herald.

[8] Araujo G, Valencia LM, Martin-Ozimek A, Soto Y, Proctor SD (2025). Atherosclerosis: from lipid-lowering and anti-inflammatory therapies to targeting arterial retention of ApoB-containing lipoproteins. Front Immunol.

[9] Slijkhuis W, Mali W, Appelman Y (2009). A historical perspective towards a non-invasive treatment for patients with atherosclerosis. Neth Heart J.

[10] Sanllorente A, Lassale C, Soria-Florido MT, Castañer O, Fitó M, Hernáez Á (2021). Modification of high-density lipoprotein functions by diet and other lifestyle changes: a systematic review of randomized controlled trials. J Clin Med.

[11] Kotlyarov S (2021). Diversity of lipid function in atherogenesis: a focus on endothelial mechanobiology. Int J Mol Sci.

[12] Akivis Y, Alkaissi H, McFarlane SI, Bukharovich I (2024). The role of triglycerides in atherosclerosis: recent pathophysiologic insights and therapeutic implications. Curr Cardiol Rev.

[13] Puri R, Bansal M, Mehta V (2024). Lipid Association of India 2023 update on cardiovascular risk assessment and lipid management in Indian patients: consensus statement IV. J Clin Lipidol.

[14] (2018). QRISK®3-lifetime cardiovascular risk calculator. https://www.qrisk.org/lifetime/.

[15] Mach F, Koskinas KC, Roeters van Lennep JE (2025). 2025 focused update of the 2019 ESC/EAS guidelines for the management of dyslipidaemias. Eur Heart J.

[16] Brandts J, Ray KK (2025). Maturation of lipid management in the 2025 ACC/AHA acute coronary syndrome guideline: what it is and what it might have been. J Am Coll Cardiol.

[17] Patel SB, Wyne KL, Afreen S (2025). American Association of Clinical Endocrinology clinical practice guideline on pharmacologic management of adults with dyslipidemia. Endocr Pract.

[18] Chrispin J, Martin SS, Hasan RK (2013). Landmark lipid-lowering trials in the primary prevention of cardiovascular disease. Clin Cardiol.

[19] Basha A, Ramakrishnan S (2024). Lipid clinical trials with special reference to Indian population. Indian Heart J.

[20] Muscoli S, Ifrim M, Russo M (2022). Current options and future perspectives in the treatment of dyslipidemia. J Clin Med.

[21] Huang JX, Yousaf A, Moon J, Ahmed R, Uppal K, Pemminati S (2025). Recent advances in the management of dyslipidemia: a systematic review. Cureus.

[22] Araújo B, Arrighini GS, Queiroga F (2025). Efficacy and safety of obicetrapib in patients with dyslipidemia: an updated meta-analysis of randomized controlled trials. Am J Prev Cardiol.

[23] (2026). Cardiovascular outcome study to evaluate the effect of Obicetrapib in patients with cardiovascular disease (PREVAIL). https://clinicaltrials.gov/study/NCT05202509.

[24] Cho L, Nicholls SJ, Nordestgaard BG (2025). Design and rationale of Lp(a)HORIZON trial: assessing the effect of lipoprotein(a) lowering with Pelacarsen on major cardiovascular events in patients with CVD and elevated Lp(a). Am Heart J.

[25] Shokravi A, Singh R, Sharma S (2025). Abstract 4369873: LPA-targeted siRNAs lower Lp(a) by over 90%: a meta-analysis of olpasiran, lepodisiran, and zerlasiran. Circulation.

[26] Nicholls SJ, Ni W, Rhodes GM (2025). Oral muvalaplin for lowering of lipoprotein(a): a randomized clinical trial. JAMA.

[27] Lewis BS (2024). First-in-human trial of PCSK9 gene editing therapy for lowering cholesterol: a new frontier in cardiovascular pharmacotherapy? News from AHA. Eur Heart J Cardiovasc Pharmacother.

